# Pressure Overload Activates DNA-Damage Response in Cardiac Stromal Cells: A Novel Mechanism Behind Heart Failure With Preserved Ejection Fraction?

**DOI:** 10.3389/fcvm.2022.878268

**Published:** 2022-06-23

**Authors:** Ilaria Stadiotti, Rosaria Santoro, Alessandro Scopece, Sergio Pirola, Anna Guarino, Gianluca Polvani, Angela Serena Maione, Flora Ascione, Qingsen Li, Domenico Delia, Marco Foiani, Giulio Pompilio, Elena Sommariva

**Affiliations:** ^1^Vascular Biology and Regenerative Medicine Unit, Centro Cardiologico Monzino IRCCS (Istituto di Ricovero e Cura a Carattere Scientifico), Milan, Italy; ^2^Department of Electronics, Information and Biomedical Engineering, Politecnico di Milano, Milan, Italy; ^3^Department of Cardiovascular Surgery, Centro Cardiologico Monzino IRCCS (Istituto di Ricovero e Cura a Carattere Scientifico), Milan, Italy; ^4^Cardiovascular Tissue Bank of Milan, Centro Cardiologico Monzino IRCCS (Istituto di Ricovero e Cura a Carattere Scientifico), Milan, Italy; ^5^Department of Biomedical, Surgical and Dental Sciences, Università degli Studi di Milano, Milan, Italy; ^6^IFOM (Istituto FIRC di Oncologia Molecolare), Milan, Italy; ^7^Department of Oncology and Hematology-Oncology, Università degli Studi di Milano, Milan, Italy

**Keywords:** heart failure, heart failure with preserved ejection fraction (HFpEF), DNA damage response (DDR), pressure overload, cardiac mesenchymal stromal cells, mechanical stretch effects

## Abstract

Heart failure with preserved ejection fraction (HFpEF) is a heterogeneous syndrome characterized by impaired left ventricular (LV) diastolic function, with normal LV ejection fraction. Aortic valve stenosis can cause an HFpEF-like syndrome by inducing sustained pressure overload (PO) and cardiac remodeling, as cardiomyocyte (CM) hypertrophy and fibrotic matrix deposition. Recently, *in vivo* studies linked PO maladaptive myocardial changes and DNA damage response (DDR) activation: DDR-persistent activation contributes to mouse CM hypertrophy and inflammation, promoting tissue remodeling, and HF. Despite the wide acknowledgment of the pivotal role of the stromal compartment in the fibrotic response to PO, the possible effects of DDR-persistent activation in cardiac stromal cell (C-MSC) are still unknown. Finally, this novel mechanism was not verified in human samples. This study aims to unravel the effects of PO-induced DDR on human C-MSC phenotypes. Human LV septum samples collected from severe aortic stenosis with HFpEF-like syndrome patients undergoing aortic valve surgery and healthy controls (HCs) were used both for histological tissue analyses and C-MSC isolation. PO-induced mechanical stimuli were simulated *in vitro* by cyclic unidirectional stretch. Interestingly, HFpEF tissue samples revealed DNA damage both in CM and C-MSC. DDR-activation markers γH2AX, pCHK1, and pCHK2 were expressed at higher levels in HFpEF total tissue than in HC. Primary C-MSC isolated from HFpEF and HC subjects and expanded *in vitro* confirmed the increased γH2AX and phosphorylated checkpoint protein expression, suggesting a persistent DDR response, in parallel with a higher expression of pro-fibrotic and pro-inflammatory factors respect to HC cells, hinting to a DDR-driven remodeling of HFpEF C-MSC. Pressure overload was simulated *in vitro*, and persistent activation of the CHK1 axis was induced in response to *in vitro* mechanical stretching, which also increased C-MSC secreted pro-inflammatory and pro-fibrotic molecules. Finally, fibrosis markers were reverted by the treatment with a CHK1/ATR pathway inhibitor, confirming a cause-effect relationship. In conclusion we demonstrated that, in severe aortic stenosis with HFpEF-like syndrome patients, PO induces DDR-persistent activation not only in CM but also in C-MSC. In C-MSC, DDR activation leads to inflammation and fibrosis, which can be prevented by specific DDR targeting.

## Introduction

According to the European Society of Cardiology (ESC) guidelines, heart failure with preserved ejection fraction (HFpEF) is a clinical syndrome caused by a structural and functional cardiac abnormality, resulting in decreased left ventricular (LV) diastolic function and diminished reserve during exercise, with normal LV ejection fraction (1). HFpEF accounts for nearly half of all HF in the developed countries (2). The population prevalence ranges from 1 to 3% and will rise further with lengthening of life expectancy and growing comorbidities. Prognosis of HFpEF is currently poor, resulting in 75% 5-year mortality and a 2.1-year median survival (3). To date, pharmacological targets to improve patient's outcomes and modify the disease natural course are not fully established (4). To reach this goal, it is crucial to gain further knowledge on the pathogenic mechanisms.

HFpEF mostly lacks a well-defined cause, typically evolving from a combination of risk factors and comorbidities, including advanced age, feminine sex, metabolic dysregulation, and hypertension (5). Pressure overload (PO) secondary to aortic valve stenosis is a well-known mechanism leading to a HFpEF-like syndrome. PO results in a compensatory remodeling mechanism of concentric LV hypertrophy (6), which, in turn, provokes increased filling pressure (7), causing HF in the long term (8). Therapeutically, aortic valve replacement is recommended in patients with severe aortic stenosis and HF symptoms, regardless of LVEF, to moderate the excessive ventricular mechanical load (9). However, not only the regression of the HFpEF-like phenotype is not always fully achieved, but it is also associated to worse long-term prognosis (10, 11).

The molecular modifications underlying PO-associated remodeling involve both the cardiac contractile compartment, resulting in cardiomyocyte (CM) hypertrophy, and the stromal compartment, showing a pro-fibrotic activation (12). In addition, HFpEF heart tissues show increased oxidative stress and inflammation (13, 14).

Recently, a link between the consequences of PO and DNA damage response (DDR) activation emerged from *in vivo* studies on an HF mouse model (15).

DNA damage activates the DDR kinases ATM and ATR, which phosphorylate multiple substrates, including H2A histone family member X (H2AX) and checkpoint proteins, CHK1 and CHK2, to orchestrate DNA repair and cell recovery. DDR-persistent activation contributes to mouse CM hypertrophy and inflammation, promoting cardiac tissue remodeling and HF (16, 17).

At the state of the art, presence and relevance of this novel molecular mechanism have not been verified on HFpEF patient samples. In addition, while a relevant role of the murine stromal compartment in the myocardial response to PO has been described (8, 18), the effects of PO on cardiac mesenchymal stromal cells (C-MSC) are still to be investigated. Indeed, C-MSC not only support cardiac structure and function in physiological conditions, but they also participate in fibrotic and inflammatory remodeling in pathological settings (19, 20).

This study addresses the aforementioned knowledge gaps, unraveling the effects of PO on DDR activation and on the subsequent DDR-driven C-MSC phenotypes switch. In light of available clinical-grade molecules targeting DDR and downstream pathway, this study opens to a novel therapeutic approach for HFpEF secondary to PO.

## Methods

### Ethics Statement

This study complies with the WMA Declaration of Helsinki and the Department of Health and Human Services Belmont Report. It was approved by “IEO-CCM IRCCS” (14/04/2021) Ethics Committee. Written informed consent was obtained from all participants. HC cardiac samples were obtained from valve donors (accidental death), from “Cardiovascular Tissue Bank” of Centro Cardiologico Monzino IRCCS (MTA signed 5 November 2019).

### Study Patient Population

A total of 7 patients with HFpEF, secondary to PO due to aortic valve stenosis, were enrolled for this study. The diagnosis was achieved, according to the ESC Consensus Recommendations (21), when the score of 5 was reached with the diagnostic algorithm. Supplementary Table S1 summarizes baseline characteristics of enrolled severe aortic stenosis with patients with HFpEF-like syndrome. We obtained LV septum samples from patients with HFpEF, undergoing aortic valve surgery, and showing concentric hypertrophy in need of septum myectomy. Patients' samples were processed within 3 h from the surgical procedure. Healthy control (HC) samples were taken from the heart of seven deceased (accidental death) valve donors. Tissue was processed within a maximum of 10 h from death (lower than the maximum time recommended by the Italian National Transplantation Center). The used septum portion would be otherwise discarded during the valve banking procedures.

### Heart Tissue Section Preparation and Immunofluorescence Analysis

Human LV septum samples were fixed in 4% paraformaldehyde (PFA) (Santa-Cruz, Dallas, TX, USA) in phosphate-buffered saline (PBS; Lonza, Basel, Switzerland) and processed for paraffin embedding. Paraffin-embedded sections (6 μm thick) were de-waxed in xylene and rehydrated in ascending alcohols. The immunofluorescence analysis was performed following antigen retrieval with incubation with target retrieval solution citrate pH6/microwave (Dako, Santa Clara, CA, USA). Sections were incubated with primary antibody anti-malondialdehyde (MDA; 1:2,500; Abcam, Cambridge, UK), anti-CD44 (1:50; Abcam, Cambridge, UK), anti-cardiac troponin T (cTNT; 1:200; Thermo Fisher Scientific, MA, USA), and anti-γH2AX (1:500; Abcam, Cambridge, UK) at 4°C overnight. After washing, sections were incubated with the appropriate fluorochrome-conjugated secondary antibody (1:200; Alexa Fluor, Waltham, MA, USA) for 1 h at room temperature (RT) in the dark. Staining for cell membrane was obtained by incubation for 1 h at RT in the dark with wheat germ agglutinin (WGA) Alexa Fluor 594 conjugated (1:200; Thermo Fisher Scientific, MA, USA). Nuclear staining was performed by incubating sections with Hoechst 33342 (1:1,000; Thermo Fisher Scientific, MA, USA). Sections were observed using Zeiss Axio Observer.Z1, with Apotome technology, and images were acquired with the AxioVision Rel. 4.8 software. For each subject, three consecutive slices and at least five fields for each slice were examined, excluding autofluorescence and aspecific signals. As quantitative indicator of CM hypertrophy, we used cell area, calculated by automated software-based image analysis (ImageJ). CM were identified by cTNT staining and, to minimize the errors introduced by the relative position between CM longitudinal axis and section plane, only CM with a circularity factor major than 0.67 (CM area HC: 0.83 ± 0.06, HFpEF: 0.83 ± 0.07, avg ± SD) were considered for the analysis. Antibody list is summarized in Supplementary Table S2.

### Hematoxylin and Eosin Staining

Dewaxed and rehydrated paraffin-embedded sections were stained with filtered 0.1% hematoxylin for 2 min and then rinsed in running distilled water for 5 min. The sections were stained in eosin (0.5% in 95% EtOH) for 4 min and rinsed again in running distilled water for 5 min. Sections were observed using Axioskop II microscope (Zeiss).

### Picro Sirius Staining

Collagen staining was performed with Picro Sirius. Dewaxed and rehydrated paraffin-embedded sections were stained with picro Sirius red solution for 1 h at room temperature. The slides were rinsed quickly in acetic acid solution twice and then dehydrated in 100% EtOH. Sections were observed using Axioskop II microscope (Zeiss).

### Masson Trichrome Staining

To stain fibrotic tissue in histological samples, Masson's trichrome staining kit (Bio-Optika) was exploited. Dewaxed and rehydrated paraffin-embedded sections were stained following the product datasheet, treating in parallel all control and patient samples. After removing the excessive colorant, the slices were dehydrated through ascending alcohols and cleared in xylene. Sections were observed using Axioskop II microscope (Zeiss), and the collagen content of the whole section was quantified as the ratio between the fibrotic area (blue staining) and the tissue area (Axiovision, Zeiss).

### TUNEL Assay

To detect apoptotic cells in HC and HFpEF total cardiac tissue, terminal deoxynucleotidyl transferase dUTP nick end labeling (TUNEL) assay was performed using the *in situ* cell death detection kit (Roche) following the instructions of the manufacturer. Briefly, dewaxed and rehydrated paraffin-embedded sections were fixed in 4% PFA for 15 min, then proteinase K solution was added and incubated at 37°C for 30 min. After 10 min of incubation with the equilibration buffer, the reaction mixture was added for 60 min at 37°C. Incubation for 10 min with Hoechst 33342 (1:1,000; Thermo Fisher Scientific, MA, USA) served for nuclei staining. For each subject, three consecutive slices and at least five fields for each slice were examined, and the percentage of TUNEL-positive cells was calculated on total nuclei.

### C-MSC Isolation and Culture

Cardiac stromal cells were isolated, and cultured as previously reported (22–25). Briefly, ventricular samples were washed with PBS, cut into 2–3 mm pieces, and incubated at 37°C for 1.5 h under continuous agitation in Iscove's modified Dulbecco's media (IMDM; Gibco, Waltham, MA, USA) containing 3 mg/ml collagenase NB4 (Serva, Heidelberg, Germany). The digested solution was then centrifuged at 400 × *g* for 10 min, washed with PBS, and centrifuged again. The obtained pellet was resuspended in growth medium (GM), consisting of IMDM supplemented with 20% fetal bovine serum (FBS; Euroclone, Milan, Italy), 10 ng/ml basic fibroblast growth factor (R&D Systems, Minneapolis, Canada), 10,000 U/ml penicillin (Invitrogen, Carlsbad, CA, USA), 10,000 μg/ml streptomycin (Invitrogen, Carlsbad, CA, USA), and 20 mmol/L l-glutamine (Sigma-Aldrich, St. Louis, MO, USA). The cells were seeded onto uncoated Petri dishes (Corning, Corning, NY, USA). Nonadherent cells were removed after 24 h. Before use for the described *in vitro* experiments, cells were expanded in culture for 4–6 passages.

### C-MSC Immunofluorescence Analysis

C-MSC were plated on 1.8 cm^2^ chamber slides (Thermo Fisher Scientific, Waltham, MA, USA) at a density of 20,000 cells/cm^2^. After 24 h of culture in basal conditions, C-MSC were washed with PBS and fixed in 4% PFA in PBS. After the blocking step in 10% goat serum (Sigma-Aldrich, St. Louis, MO, USA), cells were incubated with primary antibody anti-γH2AX (1:500; Abcam, Cambridge, UK) at 4°C overnight. After washing, sections were incubated with the appropriate fluorochrome-conjugated goat anti-rabbit antibody (1:200; Alexa Fluor, Waltham, MA, USA) for 1 h at RT in the dark. Nuclear staining was performed by incubating sections with Hoechst 33342 (1:1,000; Life Technologies, Carlsbad, CA, USA). Sections were observed using Zeiss Axio Observer.Z1, with Apotome technology, and images were acquired with the AxioVision Rel. 4.8 software. For each dish, 15 fields were examined. Antibody list is summarized in Supplementary Table S2.

### β-Galactosidase Assay

We evaluated senescence in cells isolated from HC and HFpEF-like syndrome tissues by performing a senescence-associated β-galactosidase assay (Cell Signaling Technology, MA, USA) following instructions of the manufacturer. Briefly, cells isolated from 7 HC and 7 HFpEF were seeded to reach 80% confluence on 24-well plate, in duplicate, and kept in culture for 24 h. After fixation, cells were incubated overnight with β-galactosidase staining solution. Five images per each well were acquired using AxioVert microscope (Zeiss) to quantify the percentage of β-galactosidase-positive cells on total cells.

### Western Blot Analyses

Total proteins from LV septum samples or isolated C-MSC were obtained by Laemmli lysis buffer. After quantification with DC protein assay (Bio-Rad, Hercules, CA, USA), proteins were run on SDS-PAGE gel (NUpage precast 4–12%; Invitrogen, Carlsbad, CA, USA) and transferred to nitrocellulose membrane (Bio-Rad, Hercules, CA, USA). The membrane was blocked in 5% skimmed milk-TBS for 1 h at RT and incubated overnight at 4°C with primary antibodies (see Supplementary Table S2 for details) anti-GAPDH (1:1,000; GE Healthcare, Chicago, IL, USA), anti-γH2AX (1:1,000; Abcam, Cambridge, UK), anti-pCHK1 (1:100; Cell Signaling), anti-pCHK2 (1:1,000; Cell Signaling), anti-αSMA (1:1,000; Sigma-Aldrich), and anti-TGFβ (1:1,000; Abcam, Cambridge, UK), anti-COL1A1 (1:1,000 Cell Signaling). After washes, the membrane was incubated for 1 h at RT with the appropriate HRP-conjugated secondary antibody goat anti-rabbit or goat anti-mouse (GE Healthcare, Chicago, IL, USA). Blots were washed and developed with the ECL system (Bio-Rad, Hercules, CA, USA). Images were acquired with the ChemiDoc™ Touch Imaging System (Bio-Rad, Hercules, CA, USA), and densitometric analysis was performed using the Image Lab software (Bio-Rad, Hercules, CA, USA). Data are normalized expressing as 1 the comparison group to highlight the fold differences between different groups or treatment. Antibody list is summarized in Supplementary Table S2.

### Gene Expression Analyses

Gene expression was assessed *via* quantitative real-time polymerase chain reaction (qRT-PCR). Total RNA extracted using TRIzol reagent was treated with DNase I (Ambion) and reversely transcribed using Superscript III reverse transcriptase (Invitrogen). Primer sequences are reported in Supplementary Table S3. Each sample was analyzed in duplicates with each primer pair, using 10 ng of cDNA, with CFX96 Touch Real-Time PCR Detection System (Bio-Rad; Hercules, CA, USA) using iQ SYBR Green Supermix (Bio-Rad; Hercules, CA, USA). Threshold cycles were normalized against the expression of the housekeeping gene GAPDH (ΔCt). Primer sequences are reported in Supplementary Table S3.

### Stretching Procedure

Cardiac pressure overload was simulated *in vitro* using a dynamic cell stretching bioreactor (FlexCell^®^). HC C-MSC (*n* = 6 cell lines) were seeded (30,000 cells/cm^2^) onto FlexCell silicone-bottomed dedicated 6-well plates pre-coated with 0.01 mg/ml fibronectin (Sigma-Aldrich). Then, 12 h after cell seeding, plates were assembled in the system and stimulated for 72 h, with cyclic (1 Hz) unidirectional 10% stretch (“dynamic”) in incubator (26). Identical silicone-bottomed plates were kept in culture for 72 h, without mechanical stimulation, to serve as “non-stretched” control. Stretching effects were evaluated by immunofluorescence, Western blot, and secretome analyses.

### Nuclei Circularity Evaluation

The geometrical effect of the application of the dynamic stretch was evaluated by automated analysis of nuclei shape (ImageJ). The round factor (defined as minor_axis/major_axis), calculated for cell nuclei (ranging between 25 and 177 cells per conditions) of six independent donors under dynamic stretch was compared with static condition.

### C-MSC Secretome Analysis

To analyze which molecules are secreted by C-MSC after mechanical stimulation with stretching, we used a custom multi-analyte Magnetic Luminex Assay kit (R&D systems; Minneapolis, MN, USA). The protocol recommended by manufacturers was observed, and the absorption was determined with Luminex xMAP technology (Bio-Rad Bio-Plex^®^, Hercules, CA, USA). Results were interpreted by constructing a dose/response curve according to the standards provided in the kit.

### C-MSC Treatments

A total of 30,000 cells/cm^2^ HC C-MSC (*n* = 3 cell lines) were seeded onto FlexCell silicone-bottom dedicated 6-well plates, previously coated by 0.01 mg/ml fibronectin (Sigma-Aldrich), in culture medium supplemented or not with 5 mmol/L AZ20 (Merck Life Science), an ATR inhibitor, or 5 mmol/L AZD1056 (Merck Life Science), an ATM inhibitor, for 72 h. They were subjected or not to the stretching procedure described above. Stretching effects on γH2AX, pCHK1, pCHK2, and αSMA expression were evaluated by Western blot.

### Statistical Analysis

Continuous variables were reported as mean ± standard error (summarized in Supplementary Tables S4, S5). Comparisons between normally distributed groups were performed using either paired or unpaired two-tailed Student's *t*-tests. The *n* indicated in each figure legend corresponds to biological replicates. Statistics were performed using the GraphPad Prism software. Results were considered statistically significant for *p* values < 0.05.

## Results

### HFpEF-Like Syndrome Total Cardiac Tissue Shows DNA Damage and DDR Activation

To confirm that the patient selection was appropriate, we analyzed LV septum tissues from our cohort of patients with HFpEF-like syndrome (Supplementary Table S1) and HC. According to the literature, we observed enlarged nuclei and hypertrophic cardiac fibers, higher deposited collagen content, higher oxidative stress (marked by MDA), and apoptosis (detected by TUNEL assay) in HFpEF-like syndrome samples (Supplementary Figure S1). Cardiomyocyte hypertrophy in patient samples was evident, as demonstrated by their transversal area, and myofibroblasts abundance was supported by αSMA protein expression (Supplementary Figure S1).

**Figure 1:**
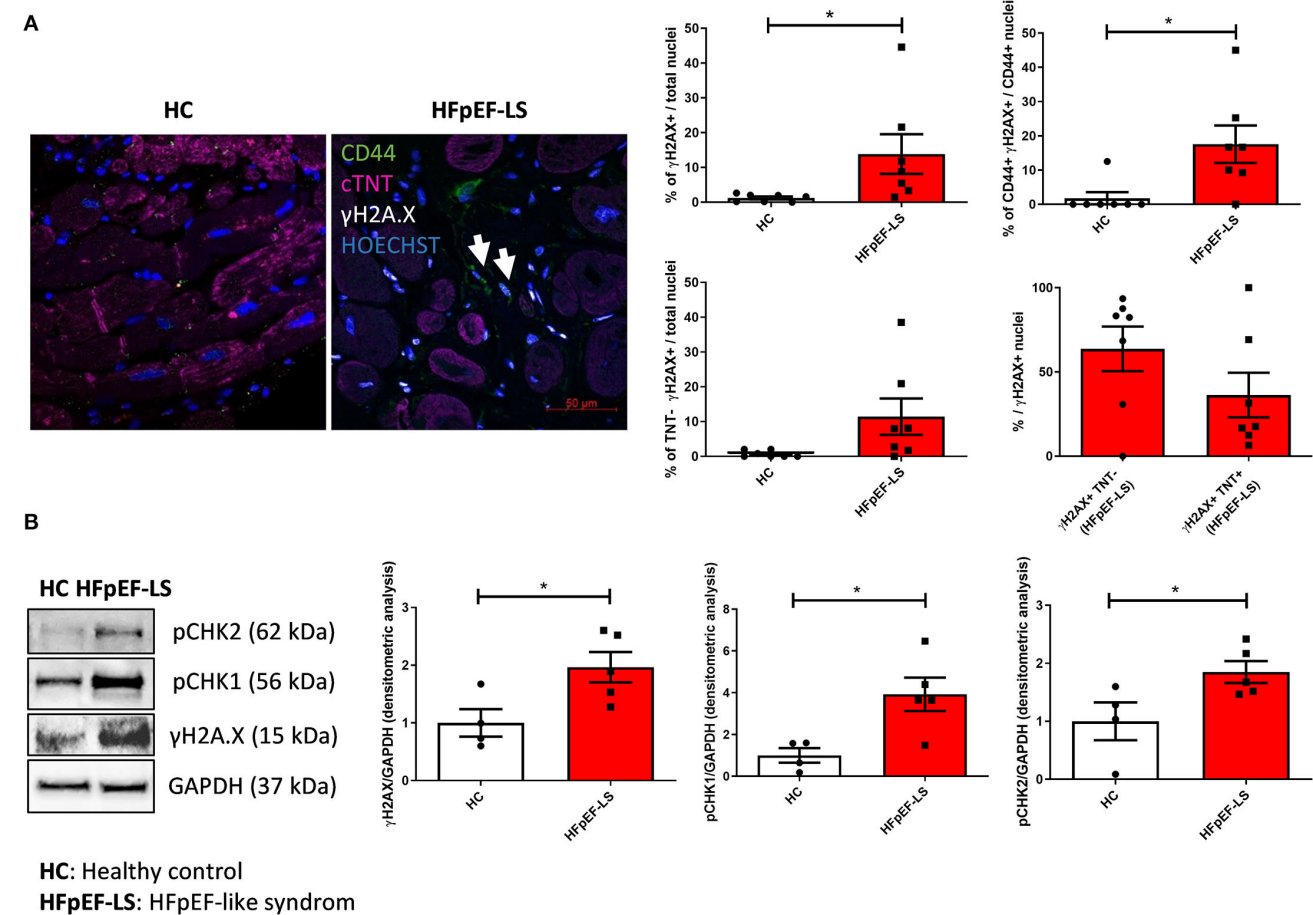
Aortic valve stenosis with HFpEF-like syndrome cardiac biopsies exhibit DDR activation. **(A)** Representative IF images of γH2AX (white), CD44 (green), and cTNT (pink) of HFpEF and HC cardiac tissue sections. Nuclei are counterstained with Hoechst 33342 (blue). Arrows indicate cells double positive for CD44 and γH2AX. The left panels show the percentages of cells positive for γH2AX, the percentages of C-MSC (positive for CD44) expressing γH2AX, the percentages of non-cardiomyocytes (cTNT negative) expressing γH2AX, and the comparison between percentages of cardiomyocytes (cTNT positive) vs. non-cardiomyocytes (cTNT negative) among the cells positive for γH2AX. ^*^ *p* < 0.05. **(B)** Western blot analysis (left) of γH2AX, pCHK1 and pCHK2 in HFpEF and HC cardiac tissue extracts. The housekeeping GAPDH is shown for normalization. Graphs on the right panels: densitometric analyses of γH2AX, pCHK1, and pCHK2 levels, normalized on GAPDH. ^*^p < 0.05.

We performed immunofluorescence analyses on human HFpEF-like syndrome and HC LV septum samples to detect DNA damage and to understand which cell types are involved. Our results show higher expression of the DNA damage marker γH2AX in HFpEF-like syndrome tissues (Figure 1A). Interestingly, the DNA damage is not confined only to cardiomyocytes, but it affects also non-cardiomyocyte cells, including C-MSC, marked by CD44. Indeed, the amount of γH2AX-positive cells is not statistically different among cardiomyocytes and non-cardiomyocytes, indicating the involvement of both the cell types in this process (Figure 1A). We confirmed DNA damage and DDR activation in severe aortic stenosis with HFpEF-like syndrome hearts by Western blot analyses (Figure 1B).

### C-MSC Are Involved in HFpEF Pathogenesis

**Figure 2:**
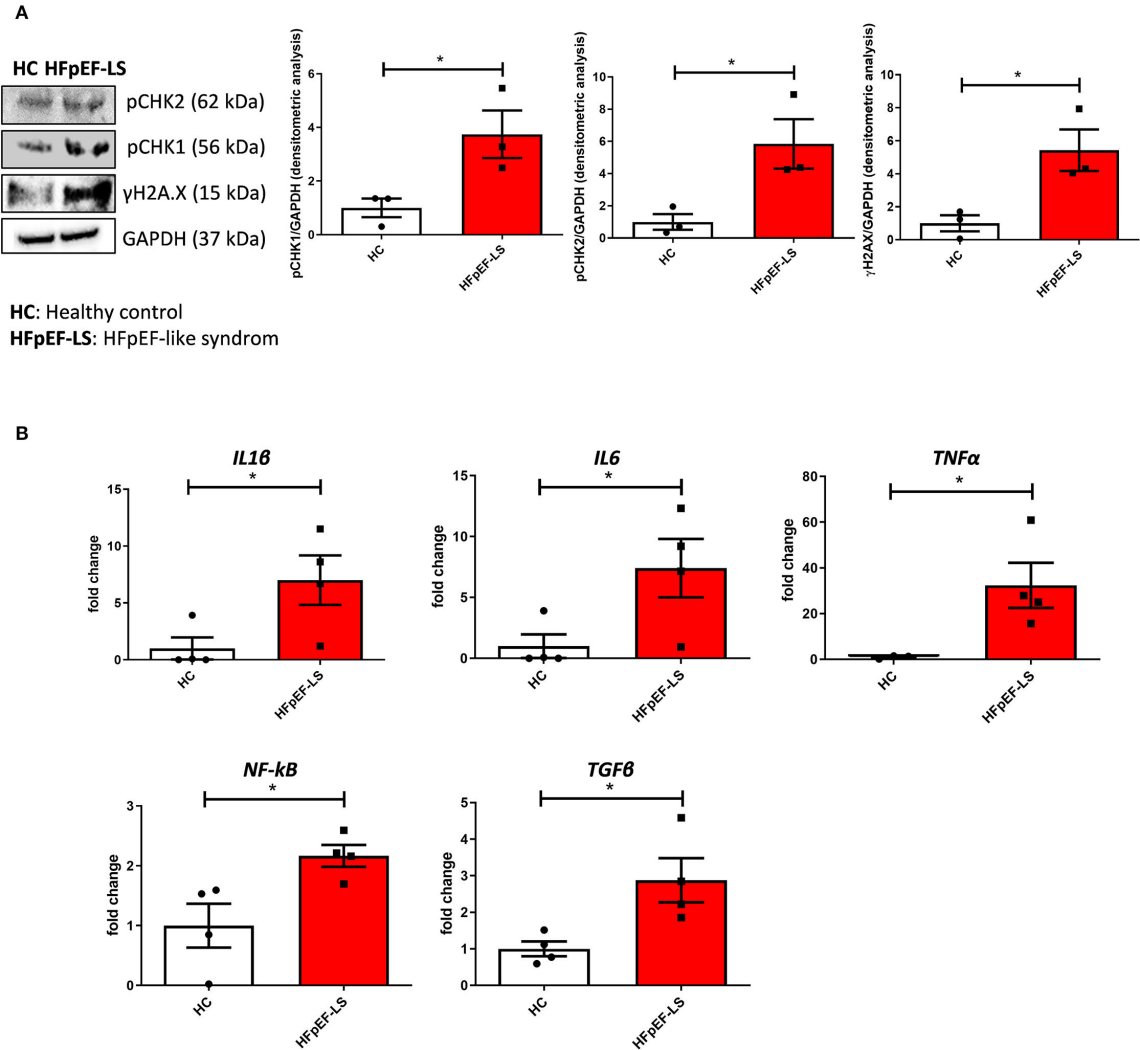
C-MSCs from patients with HFpEF-like syndrome show activated DDR and increased expression of pro-inflammatory and pro-fibrotic mediators compared to those from HC. **(A)** Left panel: Western blot analysis of anti-γH2AX, anti-pCHK1, and pCHK2 in HFpEF and HC C-MSC. The housekeeping GAPDH is shown for normalization. Right panels: densitometric analyses of γH2AX, pCHK1, and pCHK2 levels, normalized on GAPDH. ^*^p < 0.05. **(B)** Expression of inflammation- (*IL1*β, *IL6, TNF*α*, NF-kB*) and fibrosis-associated genes (*TGF*β) in total RNA extracts of C-MSC from HC donors and patients with HFpEF. GAPDH was used as a housekeeping gene, and qRT-PCR data are presented as the fold change of target gene expression with respect to HC C-MSC. ^*^p < 0.05.

As the analyses on total cardiac tissue showed DNA damage and DDR activation also in C-MSC, we performed further analysis on purified C-MSCs from HFpEF-like syndrome and HC LV septum samples, obtained as previously described (23). As expected, HFpEF-like syndrome cells express a significantly higher amount of γH2AX, pCHK1, and pCHK2 proteins than HC cells in culture conditions (Figure 2A), suggesting C-MSC involvement in DDR-driven remodeling. Importantly, β-galactosidase activity did not highlight differences between the two groups (Supplementary Figure S3), csuggesting that DDR activation is not secondary to cell senescence. To check the pro-inflammatory and pro-fibrotic potential of HFpEF-like syndrome C-MSC, we analyzed the expression of interleukin 1β (IL1β), interleukin 6 (IL6), tumor necrosis factor (TNFα), nuclear factor kappa-light-chain-enhancer of activated B cells (NF-kB), and transforming growth factor beta (TGFβ) in HFpEF-like syndrome and HC C-MSC. Notably, the expression of these pro-inflammatory and pro-fibrotic mediators and cytokines is significantly greater in HFpEF-like syndrome than HC C-MSC in culture (Figure 2B).

### Mimicking *in vitro* Pressure Overload Provokes DDR Activation in HC C-MSC

**Figure 3:**
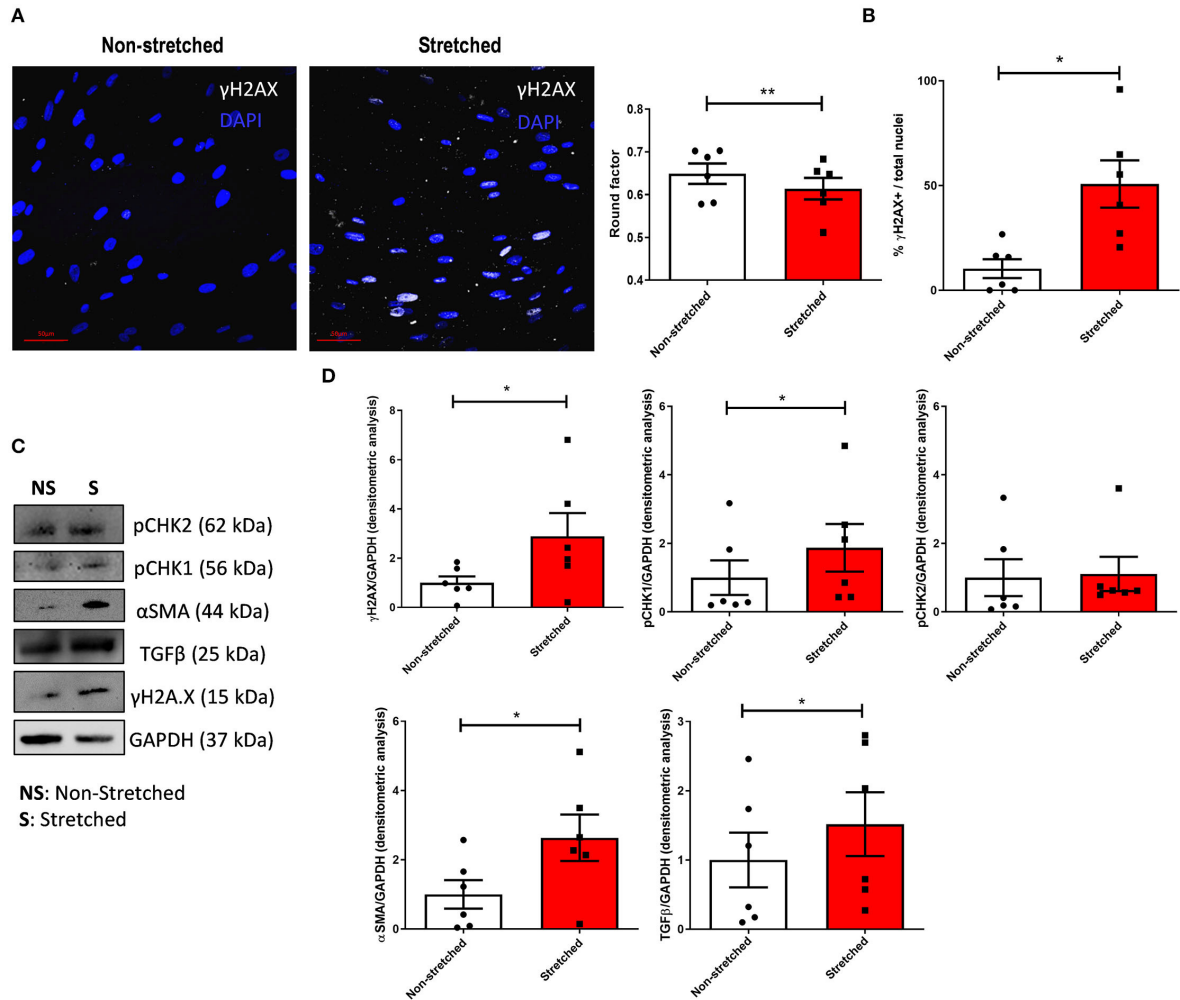
Stretch-induced mechanical stress activates the DDR in HC C-MSCs. **(A)** Representative images of γH2AX (white) immunostaining on non-stretched and stretched HC C-MSC and relative quantification. Nuclei are counterstained with Hoechst 33342 (blue). Scale bar represents 50 μm. **(B)** Automated computer-based immunofluorescence image analysis. Left: nuclei roundness factor of cells culture in static vs. dynamic conditions; right: percentage of γH2AX positive nuclei in the two culture conditions. * *p* < 0.05; ** *p* < 0.01. **(C)** Western blot analysis of γH2AX, anti-pCHK1, pCHK2, TGFβ, and αSMA in non-stretched and stretched HC C-MSC. Immunostaining of the housekeeping GAPDH is shown for normalization. **(D)** Densitometric analyses of γH2AX, pCHK1, and pCHK2, TGFβ and αSMA levels, normalized on GAPDH. *p < 0.05.

To determine if the persistent activation of DDR in C-MSC depends on PO, we subjected HC cells to cyclic unidirectional stretch (10%, 1 Hz, 72 h, FlexCell) to mimic *in vitro* the effects of PO. The effective application of the mechanical stimuli was demonstrated by a significant change in the nuclear roundness factor (Figure 3A). Stretching significantly increased the IF levels of DNA damage-associated γH2AX^+^ in C-MSC from HC Figure 3B). Accordingly, Western blot analyses confirmed the higher amount of γH2AX and pCHK1 (Figures 3C,D) in stretched C-MSC. No differences were, however, seen in pCHK2 expression (Figures 3C,D).

**Figure 4:**
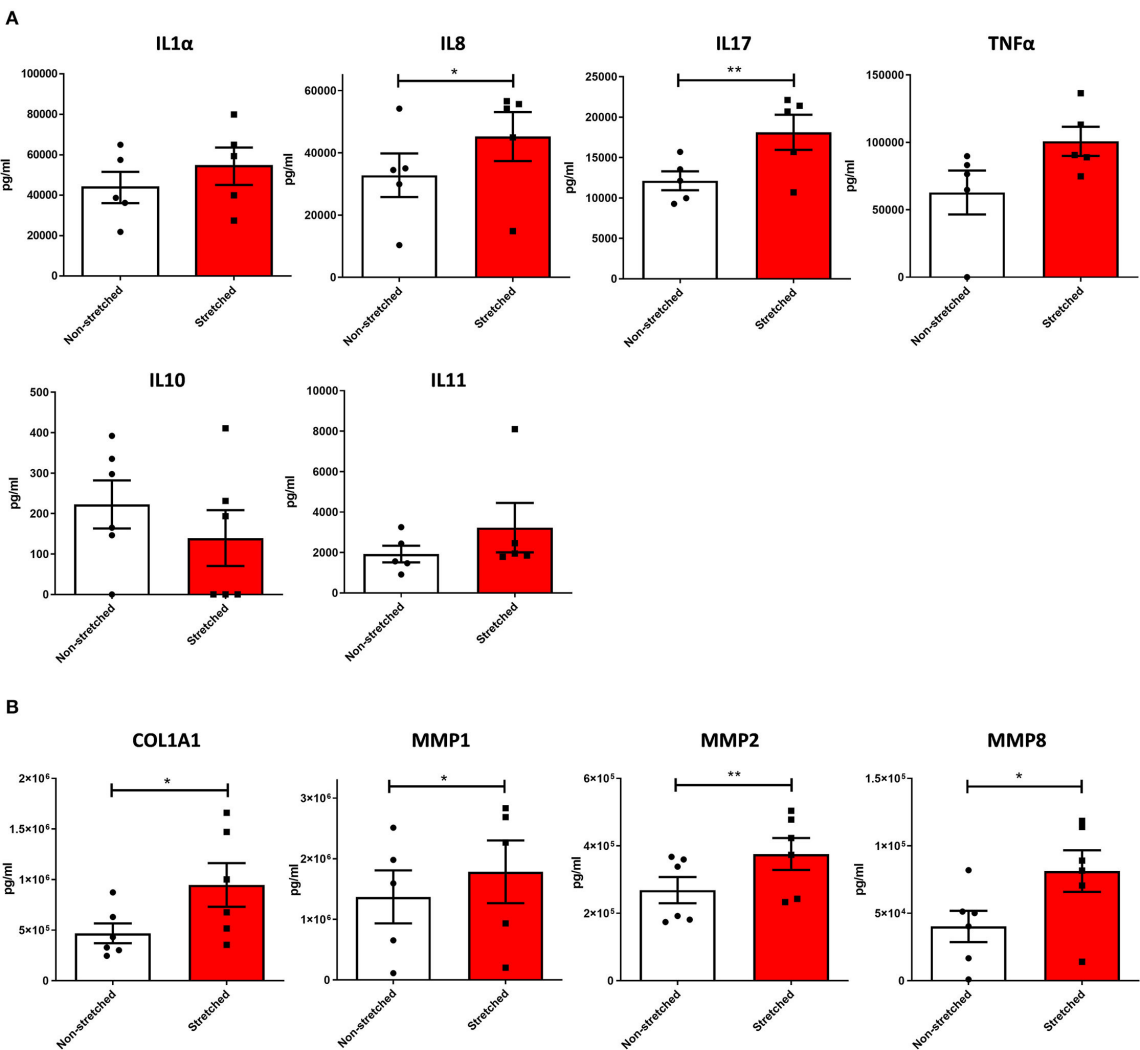
Increased secretion of pro-inflammatory and pro-fibrotic cytokines upon mechanical stretch of C-MSC. **(A)** Secretome comparison of pro-inflammatory mediators of non-stretched and stretched HC C-MSC. The concentration of each factor was calculated per ml of supernatant in 72-h medium. * *p* < 0.05; ** *p* < 0.01. **(B)** Secretome comparison of pro-fibrotic mediators of non-stretched and stretched HC C-MSC. The concentration of each factor was calculated per ml of supernatant in 72-h medium. * *p* < 0.05; ** *p* < 0.01.

To confirm if the pro-fibrotic phenotype of C-MSC depends on the mechanical non-physiological stimuli present in PO, we analyzed the expression of αSMA and TGFβ and found that their levels increased upon stretching (Figures 3C,D). Accordingly, stretched C-MSC secreted more pro-inflammatory (Figure 4A) and pro-fibrotic (Figure 4B) cytokines compared to static controls. The complete panel of secretome analytes is reported in Supplementary Table S4.

### The Pharmacological Inhibition of DDR Prevents C-MSC Pathological Response to PO

**Figure 5:**
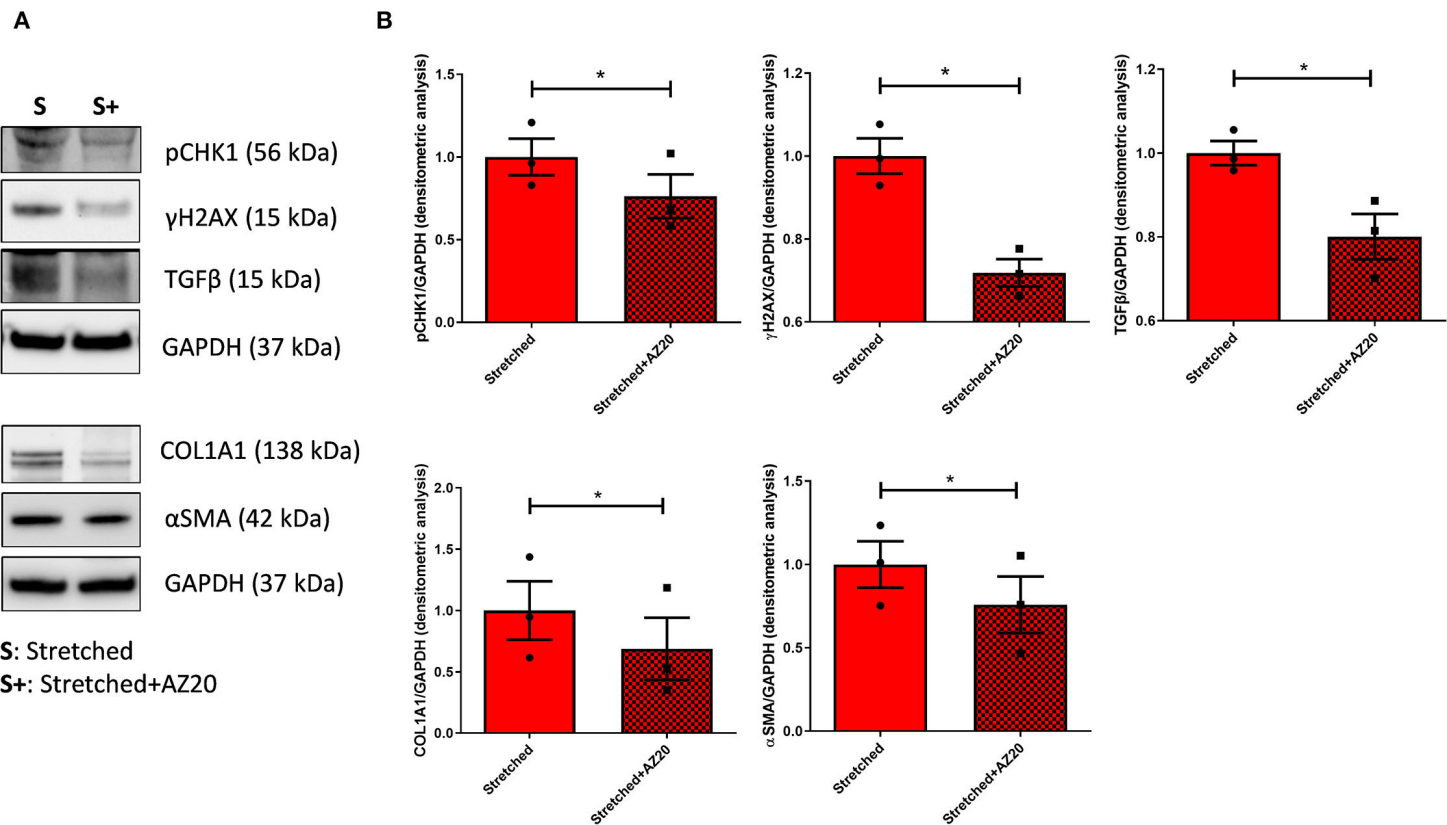
DDR inhibitor AZ20 prevents pro-fibrotic commitment. **(A)** Western blots of γH2AX, pCHK1, COL1A1, TGFβ, and αSMA from stretched HC C-MSC treated with/out AZ20. Immunostaining of the housekeeping GAPDH is shown for each gel for normalization. **(B)** Densitometric analyses of γH2AX, pCHK1, COL1A1, TGFβ, and αSMA levels, normalized on the corresponding GAPDH. * *p* < 0.05.

To understand if the modulation of PO-induced DDR activation could reduce C-MSC-associated HF pathological phenotypes, C-MSC were stretched in the presence of AZ20 5 mM, a potent inhibitor of ATR and of the Chk1 axis (27). As expected, pCHK1 signal in stretched C-MSC treated with AZ20 was significantly reduced compared to untreated stretched cells (Figure 5). Similarly, AZ20 treatment reduced γH2AX levels and it was able to diminish the pro-fibrotic markers αSMA, TGFβ, and COL1A1 (Figure 5). As expected, no differences in pCHK2, γH2AX, and αSMA expression were detected when stretched cells when subjected to treatment with 5 mM AZD0156 (*n* = 3 each; Supplementary Figure S2).

## Discussion

Despite the current standard of care, patients with HFpEF still represent an emerging burden. A better understanding of the pathogenetic mechanisms and the obtainment of new treatments is crucial to reduce the high mortality of the disease (3). The improvement in the healthcare of patients with HFpEF, by obtaining etiologic treatments, would improve disease prognosis and reduce the huge social costs of such a pandemic condition.

Toward this aim, we have addressed the impact of persistent DDR activation secondary to pressure overload in driving certain aspects of HFpEF, taking advantage of human cardiac samples from patients undergoing myectomy during aortic valve replacement and *in vitro* studies to model PO.

Previous animal studies highlighted the role of persistent DDR activation in the transition from PO-induced cardiac hypertrophy to HF following transverse aorta constriction (TAC) (15), a pathological mechanism attributed just to CM hypertrophy (16, 17). Deletion of DNA repair protein XRCC1 increased the severity of TAC-induced HF, an effect that could be rescued by deletion of ATM (17). Although TAC mouse is not universally accepted as a perfect model for HFpEF, as it also shows systolic dysfunction (28), these data indicate that PO and DDR are interconnected. In humans, the link between DDR and HF is underinvestigated, with only a few reports correlating DDR activation to cardiac dysfunction, in heart tissue from patients with end-stage cardiomyopathy (29) or myocardial infarction (30). Our studies on cardiac biopsies from severe aortic stenosis with patients with HFpEF-like syndrome further supported these findings, highlighting the involvement of both ATR/CHK1 and ATM/CHK2 axis. ATR and ATM are the apical components of the DDR responsible for the initiation and propagation of the cells' responses to DNA break (31). They induce cell cycle checkpoint activation, transcriptional feedbacks, DNA repair, and eventually apoptosis (32). In particular, transcriptional activation of sterile inflammatory-adaptive response is a typical DDR downstream event (33). Similarly, the pathological HF transcription program is enriched for pathways related to innate immune signaling converging on NFkB/RelA, and a pro-inflammatory response converging on TGFβ signaling module, leading to cellular matrix organization (34).

Our results extended the effects of DDR activation also to C-MSC, which resulted to have DNA damage even at higher proportion than CM. As C-MSC pro-fibrotic and pro-inflammatory predisposition in different pathological conditions is widely reported (19, 20), we assessed their response to mechanical stress, mimicking PO by using a mechanical stretching device, to determine the correlation between DDR and certain aspects of HFpEF-like syndrome pathogenesis. Indeed, it is known that mechanical stretch induces C-MSC proliferation and transdifferentiation into myofibroblasts (35), mainly by mechanotransduction mechanisms (26, 36). Our *in vitro* culture of human HFpEF-like syndrome-isolated cells confirmed DNA damage and DDR activation, as well as higher expression of pro-inflammatory and pro-fibrotic mediators in culture conditions, pointing to the interdependency of DDR pathway activation and C-MSC phenotypes. DDR inhibition experiments confirmed a cause-effect relationship between DDR activation and pro-fibrotic and pro-inflammatory events, thus completing the spectrum of triggers and pathways activated in stromal cells by PO.

DNA damage can be interpreted as a mere consequence of senescence and apoptosis, or an active mediator of response pathways. The persistent activation of the DDR sustained by different phenomena such as age-dependent decline in DNA repair efficiency, telomere attrition (37), and nuclear rupture contributes to apoptosis and/or senescence, and to an inflammatory state promoting cardiac tissue remodeling. In atherosclerosis, the persistent DNA damage is known to induce the senescent cells to the so-called senescence-associated secretory phenotype (with secretions of pro-inflammatory cytokines, chemokines, growth factors, and proteases), which stimulate chronic inflammation (38, 39). Furthermore, DNA damage marker predicts the severity of dilated cardiomyopathies (40).

The *in vitro*-simulated PO provoked DNA damage in HC C-MSC, leading to the upregulation of ATR/CHK1 pathway. This is not surprising since this pathway is the most frequently upregulated in pluripotent cells, like C-MSC are, in response to DNA damage. However, in the patient tissue and the harvested patient cells, both CHK1 and CHK2 kinases are phosphorylated. This means that ATM/CHK2 pathway may be activated by another trigger. Concomitantly, a higher expression of pro-fibrotic and pro-inflammatory mediators has been detected. Remodeling extent may depend on the duration of ATR/CHK1 pathway activation. The substantial direct and paracrine crosstalk between C-MSC and CM could take part in the *ex vivo* observed CM phenotypical modifications, such as hypertrophy and apoptosis (41). In particular, a direct role of inflammatory cytokines on CM contractility and mitochondrial function (42) is reported and metalloprotease regulation is implicated in hypertrophic growth of CM (43), in their contractility (44) and inflammatory signaling regulation (45). In addition, extracellular matrix modification, generated by C-MSC pro-fibrotic activation, affects cardiac tissue stiffness, generating an extra load for CM, ultimately leading to further compensatory remodeling (46, 47).

To date, HFpEF management is non-specific, including diuretics and control of causative comorbidities, such as hypertension and obesity, but the prognosis is not always favorable (9). Recently, other pharmacological strategies are emerging (5). In TAC mouse models, DDR depletion could rescue the HF phenotypes (17). The therapeutic targeting of this pathway could be effective for stromal cells of patients suffering from HF secondary to PO. Accordingly, our results provide a proof of principle of the feasibility to prevent C-MSC fibrotic phenotype through the pharmacological inhibition of the ATR/CHK1 pathway. Our data could lay the basis for future novel therapies for patients with HFpEF with specific PO-driven etiology. As the theory of a multiple (or at least double: metabolic and mechanical stress) hit is consolidating for HFpEF etiology (48), it has to be acknowledged that our results can be applied for what concern the mechanical (PO) stress due to aortic stenosis only. Indeed, it has been reported that a non-negligible proportion of patients who underwent aortic valve replacement do not experience LV hypertrophy regression (10). These no-responders show severe preoperative myocardial damage and fibrosis (49), which can potentially be targeted by DDR inhibition.

To date, the majority of the studies testing DDR inhibition for clinical purposes are in cancer field (27), and several ATR inhibitors have been proposed. AZ20 is able *in vitro* to inhibit ATR in a concentration-dependent manner, including phosphorylation of its primary substrate CHK1 (50). *In vivo* treatments with AZ20 are promising, but the translation in clinical practice is limited due to poor solubility and potential drug-drug interactions (50). An optimized version of AZ20 is available and under evaluation in phase II clinical trials for cancer treatment (27). Adverse events of ATR inhibitors mostly occur when administered in combination with cytotoxic chemotherapies, and not as monotherapies, like they would be used for patients with HFpEF (51). Further preclinical studies will better clarify the effects of DDR inhibition in HFpEF.

Some limitations of our study should be mentioned. The presented results were obtained selecting a subpopulation of patients presenting a HFpEF-like syndrome. Indeed, our cohort, while fulfilling the requirements for HFpEF diagnosis, is composed by patients with PO secondary to aortic stenosis. Despite being functional to our goal of investigating a correlation between mechanical stimulation and DDR activation, it has to be acknowledged that the obtained results cannot be directly extended to the heterogenous HFpEF scenario. Furthermore, our study includes seven patients. Despite being sufficient to provide a proof of concept on the relevance of a mechanism, an increased sample size would be necessary to support the transferability of our results. Finally, even if our HC samples (as collected from discarded cardiac tissue from valve transplantation donors) do not match our patient population for age (55 ± 2 vs. 68 ± 3 years old), we excluded through a β-galactosidase assay a major effect of senescence/aging on our results.

Further exploitation of our results could be obtained by verifying the feasibility of reverting DDR activation and the related inflammatory and fibrotic activation by treating C-MSC from patients with PO-HFpEF-like syndrome with ATM and ATR inhibitors. We are aware that the translation of such a study to the broad HFpEF population should consider HFpEF multiple etiological factors and comorbidities (e.g., metabolism).

**Figure 6:**
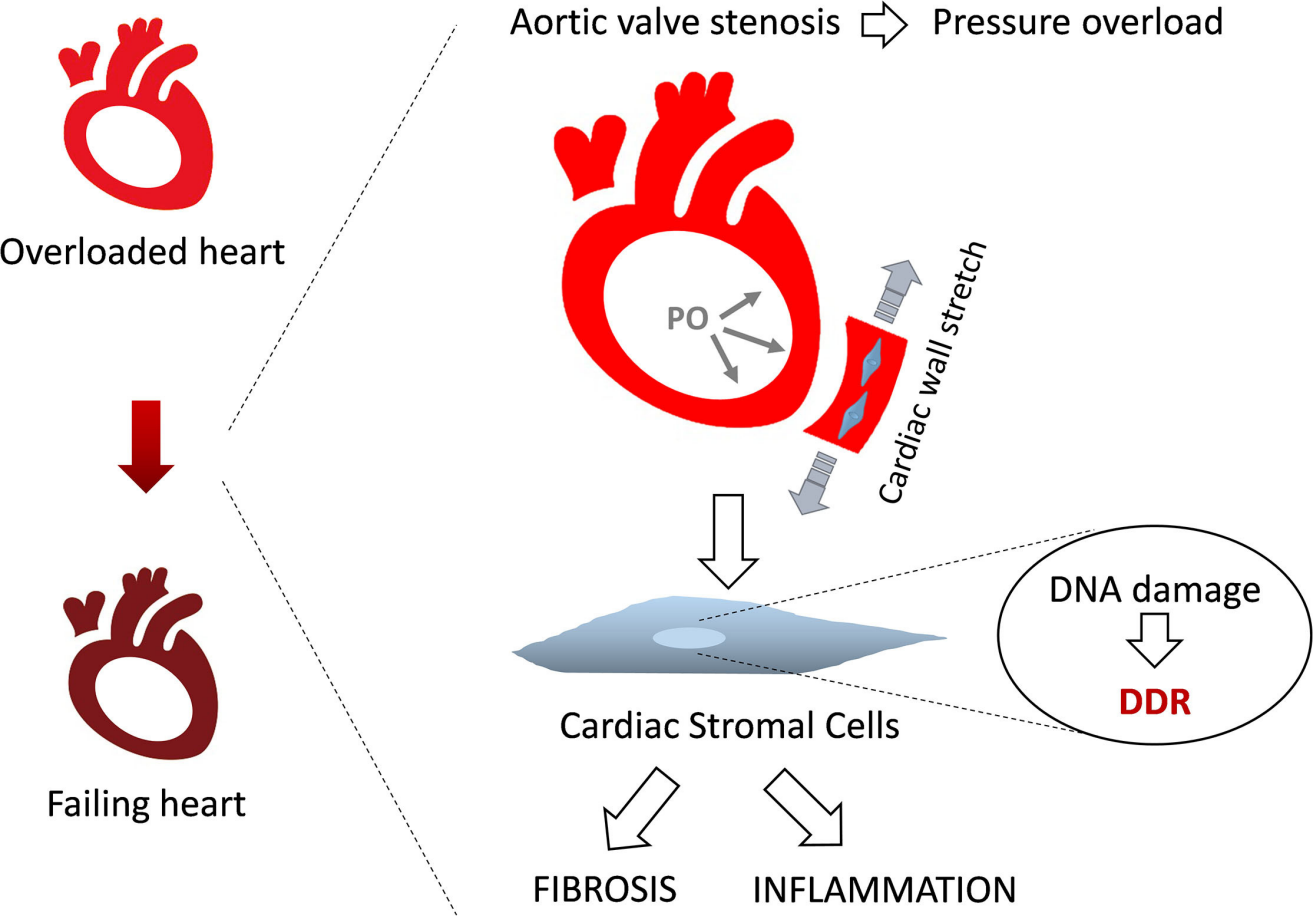
Study rationale and conclusions. Aortic valve stenosis induces cardiac pressure overload. This leads to non physiological deformation of the ventricle wall, causing DNA damage and persistent DDR activation. In turn, this promotes C-MSC pro-fibrotic and pro-inflammatory fate, contributing to heart failure degeneration.

In conclusion (Figure 6), this study highlights DDR persistent activation in both CM and C-MSC, in patients affected by HFpEF-like syndrome secondary to severe aortic valve stenosis. C-MSC isolated from samples harvested from our cohort show activation of DDR, pro-inflammatory and pro-fibrotic phenotype. Our *in vitro* experiments, mimicking the mechanical stimuli acting in PO, induced, in HC C-MSC, DDR activation and the consequent described effects, which could be reverted by ATR inhibition.

## Data Availability Statement

The datasets presented in this study can be found in the online repository ZENODO (doi: 10.5281/zenodo.6123300).

## Ethics Statement

The studies involving human participants were reviewed and approved by IEO-CCM IRCCS Ethics Committee (14/04/2021). The patients/participants provided their written informed consent to participate in this study.

## Author Contributions

IS, RS, and ES: conceptualization, experiment execution, data analysis, and original draft preparation. AS: histology experiment execution. AM: bioplex experiment execution. SP and GPol: collected clinical data. FA and QL: helped with experimental design and data interpretation; AG: provided samples from cadaveric donors. DD, MF, and GPom: handled funding and supervision. All authors have read and agreed to the published version of the manuscript.

## Funding

The project was funded by the Italian Ministry of Health—Ricerca Corrente 2764186—to Centro Cardiologico Monzino IRCCS.

## Conflict of Interest

The authors declare that the research was conducted in the absence of any commercial or financial relationships that could be construed as a potential conflict of interest.

## Publisher's Note

All claims expressed in this article are solely those of the authors and do not necessarily represent those of their affiliated organizations, or those of the publisher, the editors and the reviewers. Any product that may be evaluated in this article, or claim that may be made by its manufacturer, is not guaranteed or endorsed by the publisher.
