# Supplementary material for: Pressure Overload Activates DNA-Damage Response in Cardiac Stromal Cells: A Novel Mechanism Behind Heart Failure With Preserved Ejection Fraction?
Source: Front Cardiovasc Med. 2022 Jun 23;9:878268. doi: 10.3389/fcvm.2022.878268 (PMC9259931; doi:10.3389/fcvm.2022.878268)

Supplementary Material

Supplementary table S1. Main clinical characteristics of enrolled patients.

ID: identifier; e′: mitral annular peak early diastolic velocity; E: peak velocity of mitral inflow during early diastole; TR: Tricuspid regurgitation; PAPS: Pulmonary artery systolic pressure; LAVI: Left atrial volume index; LVEF: Left ventricular ejection fraction; LVMI: Left ventricular mass index; NYHA: New York Heart Association; hs TNI: high sensitivity Troponin I; BNP: Brain Natriuretic Peptide; n.a.: not available.

**Patient ID**	**H2**	**H3**	**H4**	**H5**	**H6**	**H7**	**H8**
**Age**	71	60	66	77	56	75	75
**Sex**	F	F	F	F	M	F	M
**Aortic valve stenosis**	yes	yes	yes	yes	yes	yes	yes
**Diabetes**	no	no	no	no	no	no	no
**Hypertension**	yes	no	yes	yes	yes	yes	yes
**Obesity**	no	no	no	yes	no	yes	no
**Atrial Fibrillation**	no	no	yes	yes	no	no	yes
**LAVI (ml/m^2)**	44	68	29	60	38	54	34
**LVMI (g/m^2)**	188	169	234	119	110	149	108
**e′ (cm/s)**	n.a.	n.a.	n.a.	n.a.	6	5	5
**E/e' ratio**	19	16	16	16	15	20	16
**TR peak velocity (m/s)**	3	3.3	2.9	3	2.9	3	3
**PAPS (mmHg)**	38	25	n.a.	n.a.	31	42	42
**LVEF (%)**	66	64	74	64	56	76	57
**NYHA class**	1	2	3	3	3	3	2
**Breathlessness**	no	yes	yes	yes	yes	yes	yes
**hs TNI (ng/l)**	7562.9	669.0	9608.3	1761.2	30619.2	13.6	n.a.
**BNP (pg/ml)**	n.a.	420	866	244	624	156	83

Supplementary Table S2. Antibody list.

Protein	Antibody	Host	Company	Dilution
MDA	ab6463	Rabbit	Abcam	IF 1:2500
CD44	ab119335	Rat	Abcam	IF: 1:50
cTNT	MA512960	Mouse IgG1	Thermo fisher scientific	IF 1:200
γH2AX	ab11174	Rabbit	Abcam	IF 1:500<br>WB 1:1000
pCHK1	#2344	Rabbit	Cell Signaling	WB 1:100
pCHK2	#2661	Rabbit	Cell Signaling	WB 1:1000
GAPDH	sc-25778	Rabbit	Santa Cruz	WB 1:1000
TGFβ	ab64715	Mouse IgG1	Abcam	WB: 1:1000
COL1A1	#84336	Rabbit	Cell Signaling	WB: 1:1000
αSMA	A 2547	Mouse IgG2a	Sigma-Aldrich	WB: 1:1000

Supplementary Table S3. Primer sequences 5’ - 3’.

Gene	Forward primer	Reverse primer
IL1β	CAAAATACCTGTGGCCTTGG	ACTGGGCAGACTCAAATTCC
IL6	ACAAAAGTCCTGATCCAGTTCC	GACTGCAGGAACTCCTTAAAGC
TNFα	AGCCCATGTTGTAGCAAACC	AGGACCTGGGAGTAGATGAGG
NF-kB	ACATCTTCCTGCTTAGTG	TCACATCTGGTTTGATTT
TGFβ1	AAGTGGACATCAACGGGTTC	GTCCTTGCGGAAGTCAATGT
GAPDH	ATGTTCGTCATGGGTGTGAA	GTCTTCTGGGTGGCAGTGAT

Supplementary table S4. Cytokines released in the culture supernatant by control C-MSC in static or dynamic culture conditions (n=6 each).

	**Non-stretched**	**Stretched**	**P value**
Adiponectin (pg/ml)	5019 ± 1618	4884 ± 836.2	0.9337
CCL4 (pg/ml)	3457 ± 1639	4357 ± 2254	0.7783
FABP4 (pg/ml)	4180 ± 509.0	3479 ± 349.7	0.2568
IL1α (pg/ml)	43843 ± 7747	54343 ± 9283	0.1553
IL6 (pg/ml)	45523442 ± 11284336	49899692 ± 11125893	0.1896
IL10 (pg/ml)	222.8 ± 59.36	139,3 ± 69.15	0.1943
IL17 (pg/ml)	12124 ± 1174	18124 ± 2174	0.0086
Leptin (pg/ml)	0	0	-
MMP2 (pg/ml)	268642 ± 38883	375696 ± 47330	0.0049
MMP9 (pg/ml)	15595 ± 1853	15235 ± 1560	0.8977
OPN (pg/ml)	695.3 ± 310.4	548,2 ± 192.3	0.3154
Thrombospondin (pg/ml)	38211 ± 12975	56742 ± 19490	0.0711
CCL2 (pg/ml)	29758667 ± 9344179	26952846 ± 9499983	0.7027
COL1A1 (pg/ml)	467619 ± 98071	946051 ± 215781	0.0141
IFNγ (pg/ml)	19736 ± 7152	10820 ± 1550	0.2825
IL1β (pg/ml)	120949 ± 13975	128866 ± 21131	0.4225
IL8 (pg/ml)	32803 ± 6984	45239 ± 7876	0.0478
IL11 (pg/ml)	1927 ± 411.8	3229 ± 1223	0.2204
IL6R (pg/ml)	337.5 ± 337.5	4400 ± 2787	0.2217
MMP1 (pg/ml)	$1.371e^{006}$ ± 437329	$1.785e^{006}$ ± 518468	0.0491
MMP8 (pg/ml)	40201 ± 11637	81293 ± 15457	0.0136
MMP13 (pg/ml)	6847 ± 651.0	6250 ± 364.7	0.4669
Resistin (pg/ml)	1583420 ± 1526729	62706 ± 3318	0.3643
TNFα (pg/ml)	62840 ± 16243	100800 ± 10825	0.1202

Supplementary table S5. Continuous variables of experiments performed.

**Fig 1**	**HFpEF-like syndrome cardiac biopsies exhibit endogenous DNA damage and DDR activation**					
		**HC**	**HFpEF**	**p**	**n (HC)**	**n (HFpEF)**
IF	% γH2AX$^+$ / total nuclei	1.23±0.41	13.87±5.71	0.05	7	7
IF	% of CD44$^+$ γH2AX$^+$ / CD44$^+$ nuclei	1.79±1.79	17.55±5.45	0.02	7	7
IF	% of TNT- γH2AX+ / total nuclei	0.70±0.36	11.43±13.83	0.06	7	7
		**TNT-**	**TNT+**		**n (HFpEF)**	**n (HFpEF)**
IF	% γH2AX$^+$ cells	63.72±13.24	36.28±13.24		7	7
		**HC**	**HFpEF**	**p**	**n (HC)**	**n (HFpEF)**
WB	γH2AX/GAPDH	1.00±0.24	1.97±0.26	0.03	4	5
WB	pCHK1/GAPDH	1.00±0.35	3.93±0.80	0.02	4	5
WB	pCHK2/GAPDH	1.00±0.33	1.85±0.19	0.05	4	5
**Fig 2**	**C-MSC are involved in HFpEF-like syndrome pathogenesis**					
		**HC**	**HFpEF**	**p**	**n (HC)**	**n (HFpEF)**
WB	γH2AX/GAPDH	1.00±0.49	5.43±1.25	0.03	3	3
WB	pCHK1/GAPDH	1.00±0.35	3.74±0.89	0.04	3	3
WB	pCHK2/GAPDH	1.00±0.49	5.85±1.53	0.04	3	3
RT PCR	*IL1β/GAPDH*	1.00±0.97	7.00±2.17	0.04	4	4
RT PCR	*IL6/GAPDH*	1.00±0.97	7.41±2.40	0.05	4	4
RT PCR	*TNFα/GAPDH*	1.00±0.43	32.38±9.85	0.04	4	4
RT PCR	*NF-kB/GAPDH*	1.00±0.37	2.17±0.18	0.03	4	4
RT PCR	*TGFβ/GAPDH*	1.00±0.20	2.88±0.60	0.03	4	4
**Fig 3**	**Stretch-induced mechanical stress activates the DDR in HC C-MSCs**					
		**Non stretched**	**Stretched**	**p**	**n (NS)**	**n (S)**
	Roundness	0.65±0.02	0.61±0.02	0.008	6	6
	% γH2AX$^+$	10.30±4.50	50.77±11.29	0.02	6	6
WB	γH2AX/GAPDH	1.00±0.26	2.88±0.94	0.05	6	6
WB	pCHK1/GAPDH	1.00±0.50	1.87±0.69	0.03	6	6
WB	pCHK2/GAPDH	1.00±0.54	1.11±0.50	0.44	6	6
WB	αSMA/GAPDH	1.00±0.41	2.63±0.67	0.03	6	6
WB	TGFβ/GAPDH	1.00±0.39	1.52±0.46	0.025	6	6
**Fig 4**	**Cytokines released in the culture supernatant by control C-MSC in static or dynamic culture conditions**					
	See Supplementay Table S4					
**Fig 5**	**DDR inhibitor AZ20 prevents pro-fibrotic commitment**					
		**Stretched**	**Stretched+ AZ20**	**p**	**n (S)**	**n (S+AZ20)**
WB	pCHK1/GAPDH	1.00±0.11	0.76±0.13	0.01	3	3

WB	γH2AX/GAPDH	1.00±0.043	0.72±0.033	0.04	3	3
WB	αSMA/GAPDH	1.00±0.14	0.76±0.17	0.02	3	3
WB	TGFβ/GAPDH	1.00±0.03	0.80±0.05	0.02	3	3
WB	COL1A1/GAPDH	1.00±0.24	0.69±0.25	0.03	3	3
**Fig S1**	**HFpEF-like syndrome patients cardiac tissue characterization**					
		**HC**	**HFpEF**	**p**	**n (HC)**	**n (HFpEF)**
WB	alfa SMA	1.00±0.12	3.21±0.19	<0.0001	7	7
Masson Trichr	fibrotic area/tissue area (%)	2.71±0.52	16.93±5.87	0.04	7	7
Tunel assay	positive nuclei/total nuclei (%)	0.86±0.17	6.59±2.35	0.0205	5	5
IF	CM area [μm2]	96.79±26.59	11450±1293	0.0001	4	4
**Fig S2**	**ATM inhibitor AZD0156 has no effect on pro-fibrotic commitment**					
		**Stretched**	**Stretched +AZD0156**	**p**	**n (S)**	**n (S+AZD0156)**
WB	γH2AX/GAPDH	1.00±0.33	1.11±0.51	0.43	3	3
WB	pCHK2/GAPDH	1.00±0.60	0.27±0.04	0.35	3	3
WB	αSMA/GAPDH	1.00±0.31	1.46±0.81	0.46	3	3
**Fig S3**	**Senescence in C-MSC derived from HC and HFpEF-like syndrome**					
		**HC**	**HFpEF**	**p**	**n (HC)**	**n (HFpEF)**
Beta Gal	% cells positive for beta gal	6.04±0.79	5.18±1.15	0.55	7	7

Supplementary Figure S1: Verification of the cardiac tissue defects in HFpEF-like syndrome patients.

A) HFpEF-like syndrome cardiac tissue is characterized by enlarged nuclei and hypertrophic cardiac fibers (Hematoxylin and Eosin staining; cTnT and WGA immunofluorescence), higher collagen deposition (Picro Sirius and Masson Trichrome staining), higher oxidative stress (malondialdehyde immunofluorescence) and apoptosis (Tunel assay), if compared to HC cardiac tissue.

B) Western blots of αSMA from total protein extract of HC and HFpEF-like syndrome cardiac tissue. Immunostaining of the housekeeping GAPDH is shown for normalization. Densitometric analyses of αSMA levels, normalized on GAPDH. *** $p<0.001$.

C) Automated computer-based immunofluorescence image analysis of fibrotic tissue (blue) on total tissue area of Masson Trichrome stained cardiac tissues.

D) Quantification of the percentage of cells positive for Tunel in the cardiac tissue.

E) Quantification of cardiomyocyte hypertrophy confirms the larger dimension of HFpEF-like syndrome cardiomyocytes than HC.

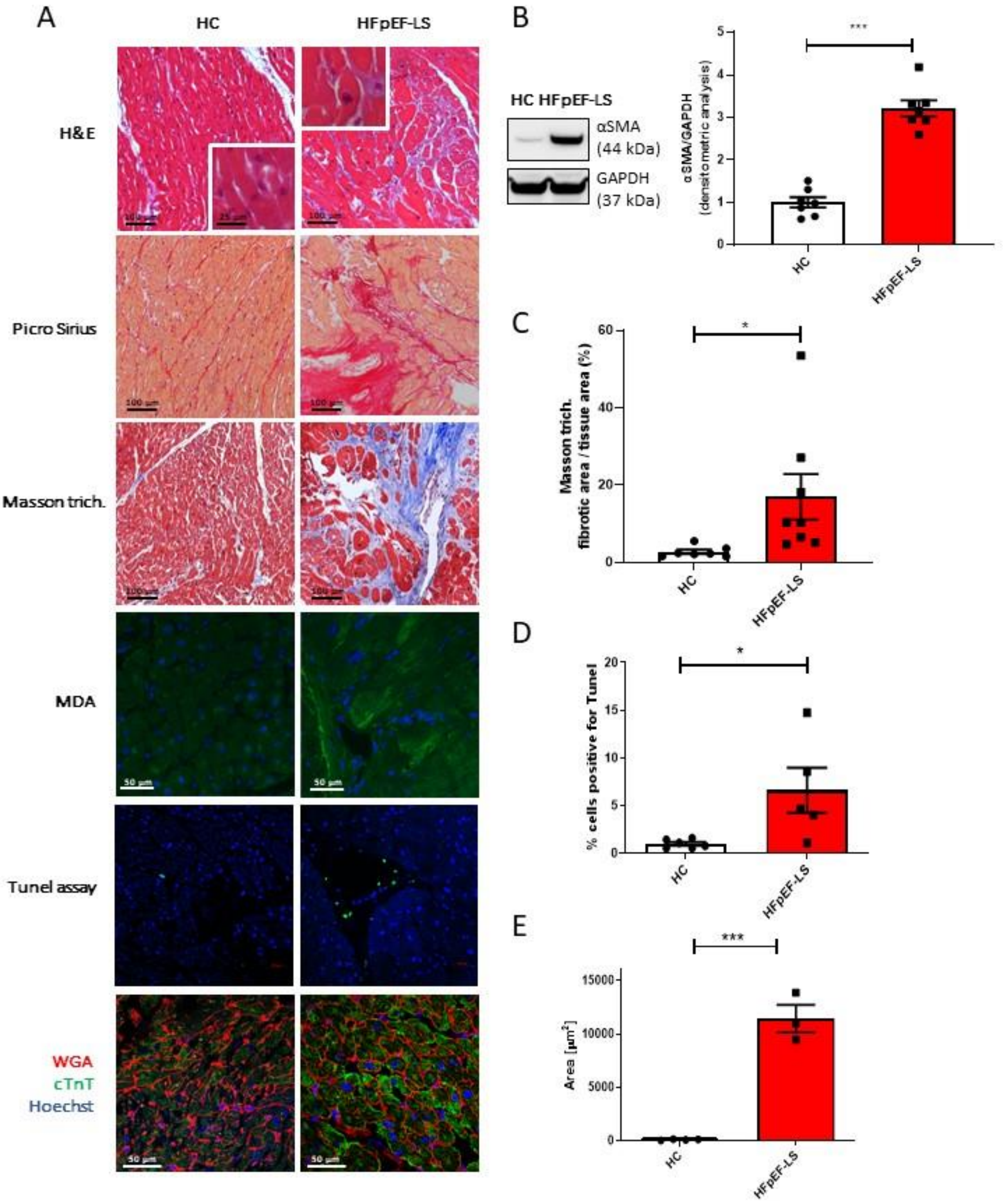
A
HC
HFpEF-LS
H&E
Picro Sirius
Masson trich.
MDA
Tunel assay
WGA
cTnT
Hoechst
B
HC HFpEF-LS
αSMA
(44 kDa)
GAPDH
(37 kDa)
αSMA/GAPDH
(densitometric analysis)
***
C
Masson trich.
fibrotic area / tissue area (%)
*
D
% cells positive for Tunel
*
E
Area [μm²]
***
HC
HFpEF-LS

Supplementary Figure S2. ATM inhibitor AZD0156 has no effect on pro-fibrotic commitment

Densitometric analyses of Western blot of proteins extracted from stretched HC C-MSC with/out treatment with 0.5 mM AZD0156, and immunoassayed for γH2AX, pCHK2, and αSMA. Protein levels, normalized on GAPDH are shown.

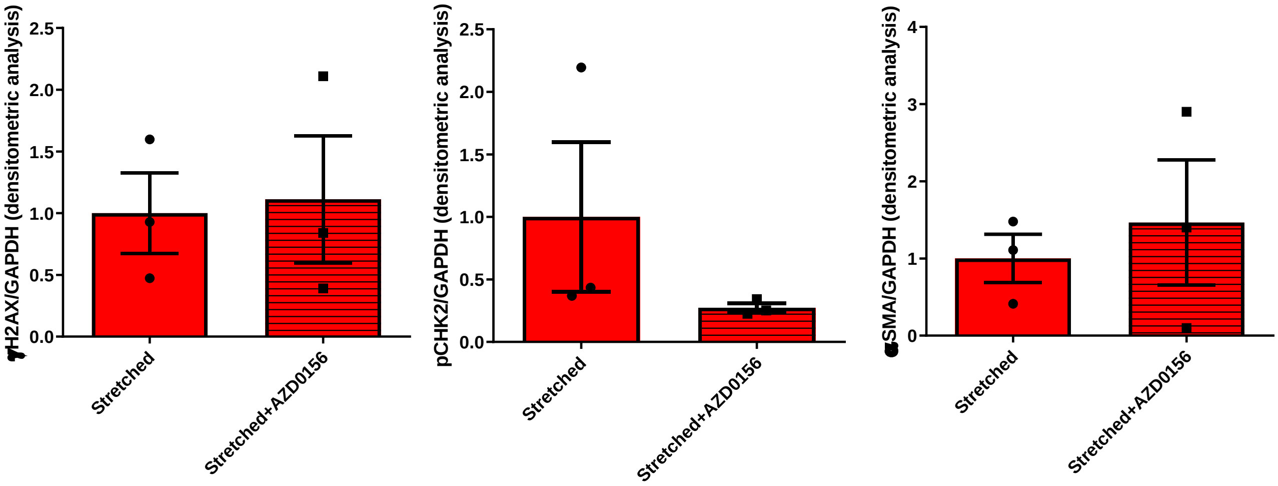

Supplementary Figure S3. Senescence in C-MSC isolated from HC and HFpEF-like syndrome samples.

Quantification of the percentage of cells positive for βgalactosidase in HC and HFpEF-like syndrome C-MSC.

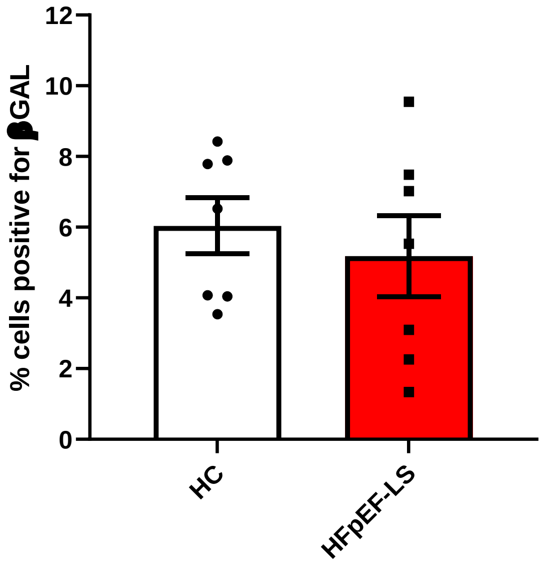

Supplement: Supplementary file 1 [file Data_Sheet_1.PDF]
