# Supplementary figures and images for: Pressure Overload Activates DNA-Damage Response in Cardiac Stromal Cells: A Novel Mechanism Behind Heart Failure With Preserved Ejection Fraction?
Source: Front Cardiovasc Med. 2022 Jun 23;9:878268. doi: 10.3389/fcvm.2022.878268 (PMC9259931; doi:10.3389/fcvm.2022.878268)

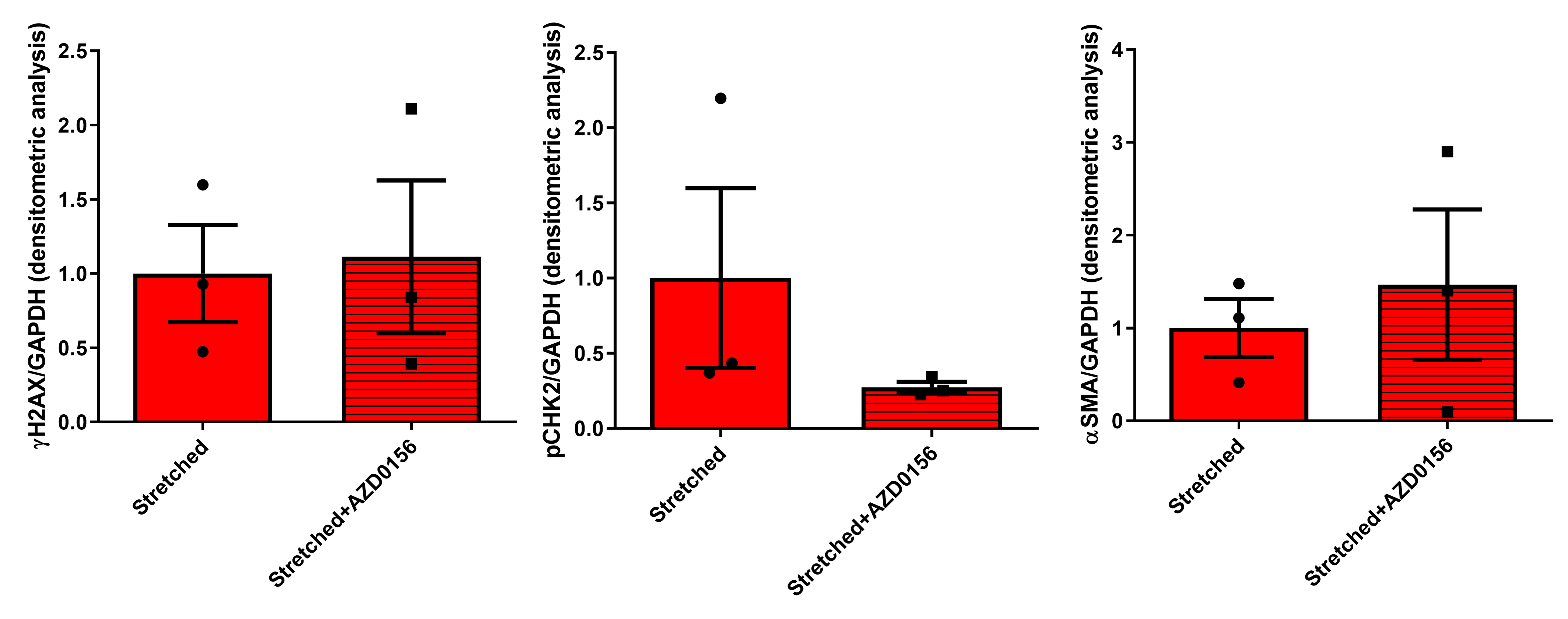

Supplement: Supplementary file 3 [file Image_2.TIF]

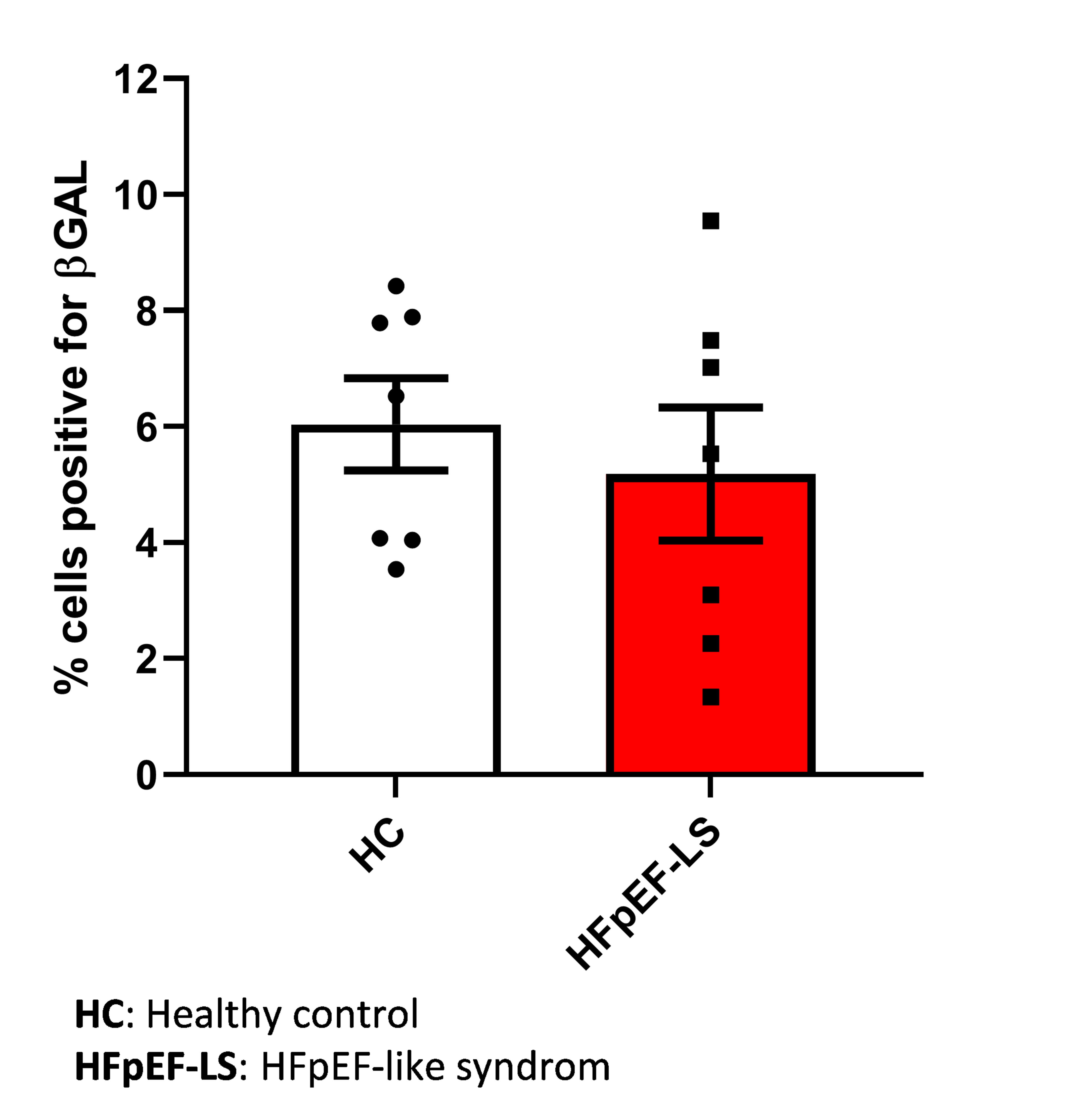

Supplement: Supplementary file 4 [file Image_3.TIF]
